# Supplementary material for: Dominant CT Patterns and Immune Responses during the Early Infection Phases of Different SARS-CoV-2 Variants
Source: Viruses. 2023 May 31;15(6):1304. doi: 10.3390/v15061304 (PMC10302336; doi:10.3390/v15061304)
Supplement: Supplementary file 1 [file viruses-15-01304-s001.zip › viruses-2427122-supplementary.pdf]

Table S1. Clinical feature of unvaccinated patients in Sixth wave (which dominant variant was Omicron)

	Omicron BA.1 without vaccine (n=24)
Age, years	56 [45-69]
Sex; number of male/female	14/10
Underlying disease	
None	5 (21)
Hypertension	8 (33)
Diabetes mellitus	3 (12)
Body mass index (kg/m^2)	24.4 [21-26]
Initial nasopharyngeal-viral load (log copies/µL)	3.9 [3.5-4.5]
RNAemia	4 (16)
Laboratory data	
Neutrophil-to-lymphoid ratio	2.6 [1.8-4.6]
LDH (IU/L)	186 [170-233]
CRP (mg/dL)	0.81 [0.3-1.9]
D-dimer (ng/mL)	0.8 [0.6-2.6]
Respiratory failure (required oxygen therapy)	3 (12)
Duration of oxygen therapy (days)	2 [1.5-2.5]
Intermittent positive pressure ventilation	0 (0)
Nasal high flow	0 (0)
Death within 30-days after onset	0 (0)

Continuous variables are reported as median [interquartile range (IQR) 25–75]. Categorical variables are reported as number (percentages).

Table S2. Radiological feature of unvaccinated patients in Sixth wave (which dominant variant was Omicron)

	Omicron BA.1 without vaccine (n=24)
CT-evaluated day from clinical onset	2.5 [1.8-3.5]
Absence of abnormal pulmonary lesion	11 (46)
Semi-quantitative CT score	2 [0-13]
Dominant CT pattern	
GGOs	11 (46)
OP	1 (4)
others	1 (4)
Range of dominant lesion	
extended over segmental area	4 (16)
within segmental area	1 (4)
within lobular area	8 (33)
Accompanied CT manifestation	
reversed halo shadow	1 (4)
curvilinear shadow	3 (12)
bronchovascular bundle thickening	2 (8)
traction bronchial dilation	0 (0)

Continuous variables are reported as median [interquartile range (IQR) 25–75]. Categorical variables are reported as number (percentages).

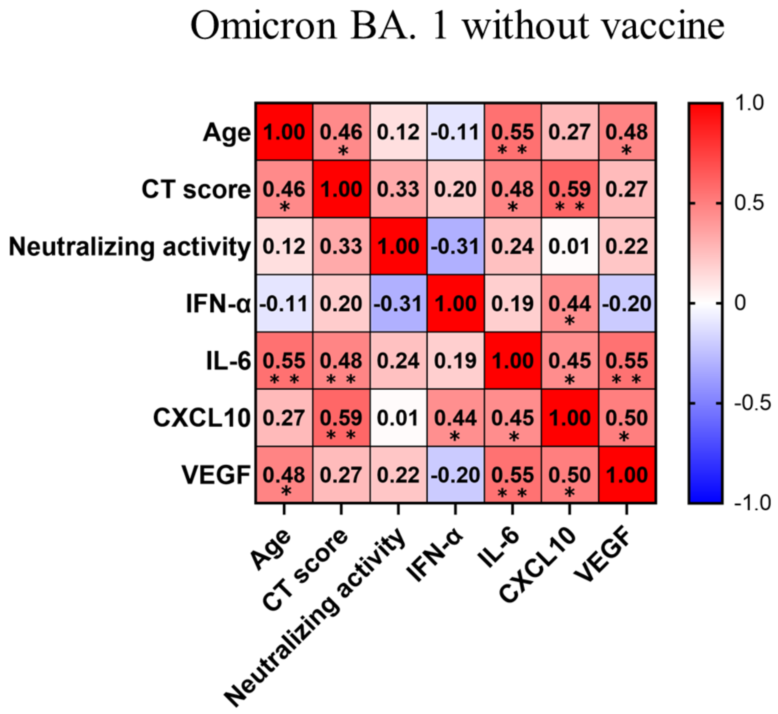

Figure S1. Correlation matrix of biomarkers in unvaccinated participants with the Omicron BA.1 variant. Each level was assessed on admission (within five days after symptom onset). Plot shows the Spearman correlation coefficients. Cells are colored according to the strength and trend of correlations (shades of red = positive correlations, shades of blue = negative correlations). ＊$p < 0.05$; ＊＊$p < 0.01$. CXCL10, C-X-C motif chemokine ligand 10; IFN, interferon; IL, interleukin; VEGF, vascular endothelial growth factor.
